# DeepNC: a framework for drug-target interaction prediction with graph neural networks

**DOI:** 10.7717/peerj.13163

**Published:** 2022-05-11

**Authors:** Huu Ngoc Tran Tran, J. Joshua Thomas, Nurul Hashimah Ahamed Hassain Malim

**Affiliations:** 1 Department of Computing, UOW Malaysia, KDU Penang University College, George Town, Penang, Malaysia; 2 School of Computer Sciences, Universiti Sains Malaysia, George Town, Penang, Malaysia

**Keywords:** Drug-target interaction, Binding affinity, Drug discovery, Deep learning, Graph neural networks, Cheminformatics

## Abstract

The exploration of drug-target interactions (DTI) is an essential stage in the drug development pipeline. Thanks to the assistance of computational models, notably in the deep learning approach, scientists have been able to shorten the time spent on this stage. Widely practiced deep learning algorithms such as convolutional neural networks and recurrent neural networks are commonly employed in DTI prediction projects. However, they can hardly utilize the natural graph structure of molecular inputs. For that reason, a graph neural network (GNN) is an applicable choice for learning the chemical and structural characteristics of molecules when it represents molecular compounds as graphs and learns the compound features from those graphs. In an effort to construct an advanced deep learning-based model for DTI prediction, we propose Deep Neural Computation (DeepNC), which is a framework utilizing three GNN algorithms: Generalized Aggregation Networks (GENConv), Graph Convolutional Networks (GCNConv), and Hypergraph Convolution-Hypergraph Attention (HypergraphConv). In short, our framework learns the features of drugs and targets by the layers of GNN and 1-D convolution network, respectively. Then, representations of the drugs and targets are fed into fully-connected layers to predict the binding affinity values. The models of DeepNC were evaluated on two benchmarked datasets (Davis, Kiba) and one independently proposed dataset (Allergy) to confirm that they are suitable for predicting the binding affinity of drugs and targets. Moreover, compared to the results of baseline methods that worked on the same problem, DeepNC proves to improve the performance in terms of mean square error and concordance index.

## Introduction

### Cheminformatics in drug discovery

The great increasing of data in the field of chemistry over the last few decades has been witnessed, as well as the data science approaches created to analyze it. In this uprising, machine learning is still the most significant tool for analyzing and understanding chemical data (Lo et al., 2018). Machine learning is a branch of artificial intelligence that targets at developing and using computational models to learn from data and to also generate more new data. Machine learning, particularly deep learning, is becoming progressively more important in operations that deal with large amounts of data, such as drug discovery.

Drug discovery is the work carried out to find the functions of bioactive compounds for the development of novel drugs, *i.e.*, searching for the candidates for new medicines (Tran et al., 2021). Traditionally, drug discovery can be considered an iterative screening process. First of all, the biochemical target is chosen; it should be known that target is a biomolecule of an organism that can be bound by a molecular structure and has its function modulated (Rifaioglu et al., 2019). The next step is to find, through chemical experiments, the compounds which interact with chosen target; these compounds are then called hits. A hit is pretty far from having an approved novel medical drug, however, it can be considered potential to be a drug candidate. Various wet-lab experiments will be performed to achieve validated hits—the hits with acceptable robustness, and then the leads—the subset of validated hits selected by parameters such as patentability and synthesizability. Many more examinations will be carried out to see which lead has physical and chemical properties such that it can be used in practice, resulting in having a smaller set called optimized leads. Finally, optimized leads will go through toxicity tests to become clinical candidates (Chan et al., 2019).

Considering the daunting tasks and large costs of the drug discovery process, computational applications become potential to be assisting tools in many stages of the process. This purpose is one of the motivations for the creation and development of cheminformatics field.

Cheminformatics is the domain where scientists adopt computer science techniques and tools to solve the problems in chemistry. It is a crucial research topic in drug discovery and drug development, since it focuses on challenges like chemical information analysis, as well as molecular data exploration. In the progressive cheminformatics, machine learning and deep learning are widely applied to method huge chemical knowledge and to get novel drugs. However, there is still an existing challenge which is that classical machine learning algorithms require data of a rigid format, *e.g.*, vectors of fixed length, which is not enough to describe chemical structures. The amount of research that requires deep learning techniques to learn important structural information of chemical data are increasing. Thus, algorithms particularly designed to handle graph data are earning more and more attention. Such algorithms are presently known as graph neural networks (GNN).

The rest of this article briefly covers the works of GNN from the beginning days until the up-to-date researches and significant applicable research works in cheminformatics. The overall architecture of the proposed model using GNN contains detailed information from the experimental work: data collecting and processing, selection of evaluation metrics, the results of training an discussion. The article ends with the concluding remarks.

### Research contributions

In this study, we introduce a deep learning-based framework for DTI prediction, which we call DeepNC. We utilized three GNN algorithms: Generalized Aggregation Networks, Graph Convolutional Networks, and Hypergraph Convolution-Hypergraph Attention, which were originally proposed in the research works by Li et al. (2020), Kipf & Welling (2017), and Bai, Zhang & Torr (2021), respectively. These algorithms play an important part for the whole model in the stage of learning the graph-based input data and creating graph-based representation of that data.

The study also presents an independent drug-target binding affinities dataset which is called the allergy dataset. The purpose of building the allergy dataset is to create a novel DTI collection that can be put in computational DTI prediction models, serving the aim of searching for new potential allergy drugs. In our scope of research, we defined allergy drugs as the approved drugs that are able to treat allergic reactions in humans; each allergy drug has one or more targets which are known as allergy drug targets. The dataset contains these allergy drug targets and the compounds that have appropriate binding affinity values towards these targets. The construction of this independent dataset will be further explained in ‘Datasets” section.

## Preliminaries

### Graph neural networks

Graphs are a form of knowledge whose structure contains a group of nodes, which represent a group of objects, and a group of edges linking them, which symbolize the objects’ relationship. In recent times, as a result of robust communicative power of graphs, analyses of data science processing graphs with machine learning are abundant and being more developed and progressed, *i.e.*, graphs may be used as representations in various areas such as social networks (Kipf & Welling, 2017; Hamilton, Ying & Leskovec, 2017), physical systems (Sanchez-Gonzalez et al., 2018; Battaglia et al., 2016), protein-protein interaction networks (Fout et al., 2017), knowledge graphs (Hamaguchi et al., 2017), and plenty of different research areas (Dai et al., 2017).

One of the most basic model of graph neural network was introduced by Scarselli et al. (2009). The framework suggests that the propagation functions be applied repeatedly until the node’s representations reach a stable state. A node *v* in the graph is characterized by its state embedding *h*_*v*_ ∈ ℝ^*D*^ and related nodes; respectively, an edge *e* is associated with its edge features *h*_*e*_ ∈ ℝ^*C*^. This GNN fundamental function is to learn and to create an embedding *h*_*v*_ for the state of the node *v*. The embedding is a vector of *b*-dimension that contains information of node *v*’s neighborhood, and is next applied to calculate and construct an output embedding *o*_*v*_.

### Graph convolution network

The architecture of Graph Convolution Network (GCN) was one of the early frameworks introduced by Kipf & Welling in the research work Kipf & Welling (2017). This graph convolution network was constructed based on the fundamental rule that says the features of each node in a message-passing layer are updated by aggregating the features of its neighbours. Kipf proposed a layer-wise propagation rule as that can be applied on a graph convolutional network of many layers. The rule is written as follow:

(1) \begin{eqnarray*}{H}^{(l+1)}=\sigma ({\widetilde {D}}^{- \frac{1}{2} }\widetilde {A}{\widetilde {D}}^{- \frac{1}{2} }{H}^{ \left( l \right) }{W}^{ \left( l \right) })\end{eqnarray*}

knowing that *H*^(^^0^^)^ = *X*, *W*^(*l*)^ represents a layer-specific trainable weight matrix, *σ*(.) is an activation function, *H*^(*l*)^ ∈ ℝ^*n*×*d*^ is the activation matrix in the *l*^*th*^ layer.

For an *n*-vertice graph, its adjacency matrix is denoted as *A* ∈ℝ^n×n^ where:

(2) \begin{eqnarray*}{A}_{ij}= \left\{ \begin{array}{@{}l@{}} \displaystyle 1 \mathrm{if} {e}_{ij}\in E\\ \displaystyle 0 \mathrm{if} {e}_{ij}\not \in E \end{array} \right. \end{eqnarray*}

and degree matrix is denoted as *D* ∈ ℝ^n×n^ where:

(3) \begin{eqnarray*}{D}_{ii}=d({v}_{i})\end{eqnarray*}

then *Ã* is computed as *Ã* = *A* + *I*_*N*_ with *I*_*N*_ is the identity matrix and ${\tilde {D}}_{ii}={\sum }_{j}{\tilde {A}}_{ij}$.

The definition of the GCN is demonstrated as:

(4) \begin{eqnarray*}Z={\widetilde {D}}^{- \frac{1}{2} }\widetilde {A}{\widetilde {D}}^{- \frac{1}{2} }X\Theta \end{eqnarray*}

where Θ ∈ℝ^*C*×*F*^ is a matrix of filter parameters with *C* input channels (*i.e.*, every node will have a C-dimensional feature vector) and *F* filters for the feature.

**Figure 1:**
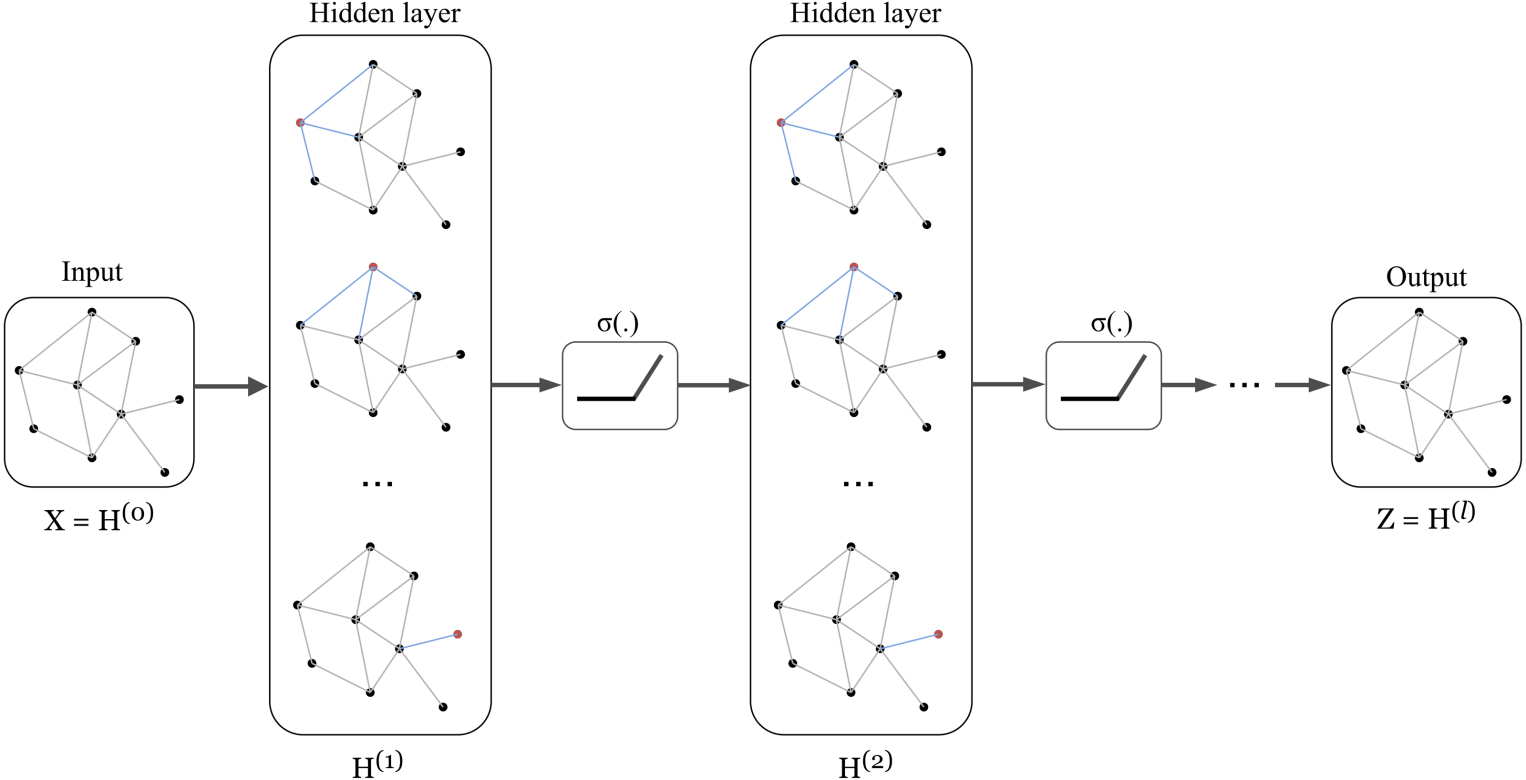
Illustration of a multi-layer graph convolutional network.

The concept of GCN can be visualized in Fig. 1 in which a graph representation is fed into convolutional networks to learn the graph output at the *l*-th layer.

### Generalized aggregation graph network

For the purpose of training deeper GCN based on the basic GCN, Li et al. (2020) created a simple *message-passing*-based GCN that meets the message-passing requirements. Firstly, at the *l*-th layer, we consider that ${m}_{v}^{ \left( l \right) }\in {\mathbb{R}}^{D}$ is node *v*’s aggregated message, ${m}_{vu}^{ \left( l \right) }\in {\mathbb{R}}^{D}$ is an individual message for each neighbor *u* ∈ *N* (*v*) of node *v*. The neighbor’s message, node *v*’s aggregated message and its features are updated as following equations:

(5) \begin{eqnarray*}{m}_{vu}^{ \left( l \right) }& ={\rho }^{ \left( l \right) }({h}_{v}^{ \left( l \right) },{h}_{u}^{ \left( l \right) },{h}_{{e}_{vu}}^{ \left( l \right) })\end{eqnarray*}

(6) \begin{eqnarray*}{m}_{u}^{ \left( l \right) }& ={\zeta }^{ \left( l \right) }({m}_{vu}^{ \left( l \right) })\end{eqnarray*}

(7) \begin{eqnarray*}{h}_{v}^{(l+1)}& ={\phi }^{ \left( l \right) }({h}_{v}^{ \left( l \right) },{m}_{v}^{ \left( l \right) })\end{eqnarray*}

in which *ρ*^(*l*)^, *ζ*^(*l*)^, and *ϕ*^(*l*)^ are learnable or differentiable functions for respectively message constructions, message aggregation, and node update at the *l*-th layer.

Li expanded Eq. (12) by defining the message construction *ρ*^(*l*)^ as follow:

(8) \begin{eqnarray*}{m}_{vu}^{ \left( l \right) }={\rho }^{ \left( l \right) }({h}_{v}^{ \left( l \right) },{h}_{u}^{ \left( l \right) },{h}_{{e}_{vu}}^{ \left( l \right) })=\text{ReLU}({h}_{u}^{ \left( l \right) }+\varphi ({h}_{{e}_{vu}}^{ \left( l \right) })\cdot {h}_{{e}_{vu}}^{ \left( l \right) })+\end{eqnarray*}

where ReLU is a rectified linear unit that outputs values to be greater or equal to 0; *φ*(.) is an indicator function giving 1 when there are edge features, and 0 for otherwise; *ɛ* is a small positive constant chosen as 10^−7^.

The message aggregation *ζ*^(*l*)^ function was proposed to be either SoftMax Aggregation or PowerMean Aggregation. In order to construct these two functions, Li also suggested a concept called Generalized Message Aggregation Function: a generalized message aggregation function *ζ*_*x*_(.) is defined as that is parameterized by a continuous variable *x* to produce a group of permutation invariant set functions. From this definition, they continued to propose the Generalized Mean-Max Aggregation: *ζ*_*x*_(.) is a generalized mean-max aggregation function if a pair of *x* = {x_1_, x_2_} exists such that for any message, *lim*_*x*→*x*_1__*ζ*_*x*_(⋅) = *Mean*(⋅) and $li{m}_{x\rightarrow {x}_{2}}{\zeta }_{x} \left( \cdot \right) =Max(\cdot )$. Given any message set *m*_*vu*_ ∈ℝ^*D*^, SoftMax Aggregation and PowerMean Aggregation are generalized functions respectively defined as:

(9) \begin{eqnarray*}SoftMax\text{_}Ag{g}_{\beta }(\cdot )& =\sum _{u\in N(v)} \frac{\exp \nolimits (\beta {m}_{vu})}{\sum _{i\in N(v)}\exp \nolimits (\beta {m}_{vi})} \end{eqnarray*}

(10) \begin{eqnarray*}PowerMean\text{_}Ag{g}_{p}(\cdot )& ={ \left( \frac{1}{ \left\vert N(v) \right\vert } \sum _{u\in N(v)}{m}_{vu}^{p} \right) }^{ \frac{1}{p} }\end{eqnarray*}

where *β* is a continuous variable called inverse temperature, and *p* is a non-zero continuous variable denoting *p*-th power.

Finally, in the phase of node update, Li applied a message normalization layer to the node update function, hence the function Eq. (14) became:

(11) \begin{eqnarray*}{h}_{v}^{(l+1)}={\phi }^{ \left( l \right) }({h}_{v}^{ \left( l \right) },{m}_{v}^{ \left( l \right) })=MLP \left( {h}_{v}^{ \left( l \right) }+s\cdot { \left\| {h}_{v}^{ \left( l \right) } \right\| }_{2}\cdot \frac{{m}_{v}^{ \left( l \right) }}{{ \left\| {m}_{v}^{ \left( l \right) } \right\| }_{2}} \right) \end{eqnarray*}

where *MLP* (⋅) is a multi-layer perceptron, and s is a learnable scaling factor. In practice, s is set to be a learnable scalar with an initialized value of 1.

### Hypergraph convolution and hypergraph attention

Most existing studies on GNNs consider the graphs as simple graphs, *i.e.*, each edge in a graph only connects two nodes. To describe more complicated graph structure in practical applications, the concept of hypergraph, the case where one edge can link more than two nodes (vertices), has been further studied. In this case, we can consider ***G*** = (***V***, ***E***) as a hypergraph of *n* nodes and *m* hyper-edges. Each hyper-edge *e* ∈ ***E*** is presented by a positive weight value *W*_*ee*_ and all the weights build up a diagonal matrix *W* ∈ℝ^*m*×*m*^. While an adjacency matrix *A*, as defined by (9), is used to represent a simple graph, it is an incidence matrix *H* ∈ℝ^*n*×*m*^ that is employed to represent the hypergraph ***G***: the element *H*_*ie*_ of *H* is 1 when the hyper-edge *e* has a link to node *v*_*i*_, otherwise it is 0.

**Figure 2:**
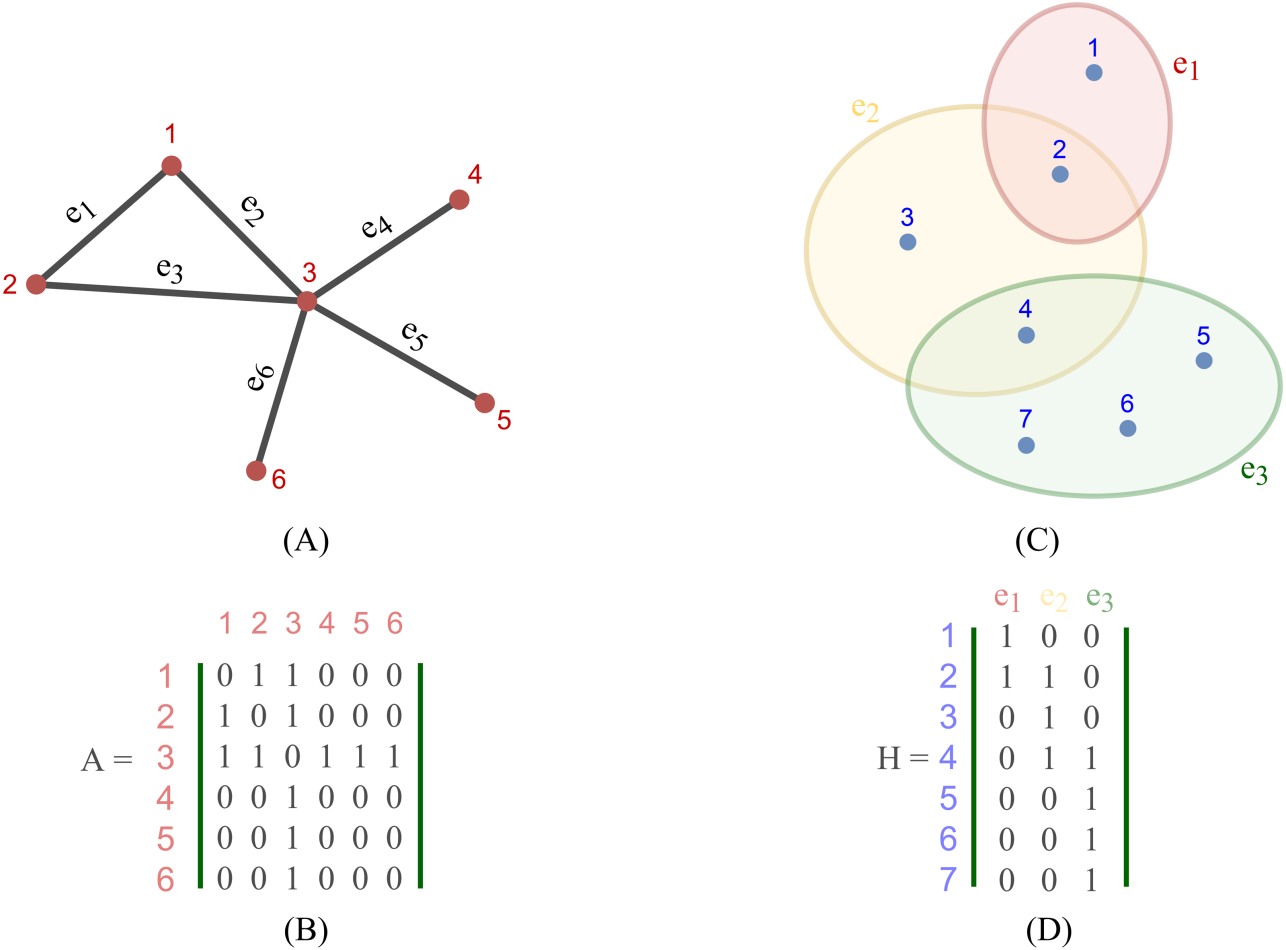
Depiction of a simple graph and a hypergraph. (A) A simple graph of six nodes and six edges (B) The adjacency matrix of the simple graph (A) (C) A hypergraph of seven nodes and three hyper-edges (D) The incidence matrix of the hypergraph (C).

Figure 2 depicts the visual distinction between a simple graph and a hypergraph, which are (A) and (C), respectively. Each edge of (A), shown by a line, simply connects two nodes in a basic graph. Each hyperedge of (C), marked by a colored ellipse in a hypergraph, connects more than two vertices. Matrix (B) and (D) are respectively the representation form of the simple graph (A) and the hypergraph (C).

In terms of methodology, hypergraph convolution approximates each hyperedge of the hypergraph with a set of pairwise edges connecting the hyperedge’s nodes, and the learning issue has been treated as a graph-learning problem on the approximation.

To define a convolution in a hypergraph, Bai, Zhang & Torr (2021) assumed that more propagation functions should be applied on the nodes linked by a common hyperedge, and the hyperedges with larger weights deserve more confidence in such a propagation. The proposed the hypergraph convolution is demonstrated as:

(12) \begin{eqnarray*}{x}_{i}^{(l+1)}=\sigma \left( \sum _{j=1}^{n}\sum _{i=1}^{m}{H}_{ie}{H}_{je}{W}_{ee}{x}_{j}^{ \left( l \right) }P \right) \end{eqnarray*}

where ${x}_{i}^{(l)}$ is the embedding representation of node *v*_*i*_ in the *l*-th layer, *σ* is a non-linear function which can be, for example, eLU (Clevert, Unterthiner & Hochreiter, 2016) and LeakyReLU (Maas, Hannun & Ng, 2013), and *P* is the weight matrix between *l*-th and (*l+* 1)-th layer. Equation (12) can also be described in a matrix form as:

(13) \begin{eqnarray*}{X}^{(l+1)}=\sigma ({HWH}^{\mathrm{T}}{X}^{(l)}P).\end{eqnarray*}

Bai also stated that stacking multiple layers like the operator Eq. (20) could result in numerical instabilities and a possible risk of vanishing gradients in a neural networks. Hence, a symmetric normalization was imposed on Eq. (19) and made it become:

(14) \begin{eqnarray*}{X}^{(l+1)}=\sigma ({D}^{- \frac{1}{2} }HW{B}^{-1}{H}^{\mathrm{T}}{D}^{- \frac{1}{2} }{X}^{ \left( l \right) }P)\end{eqnarray*}

where *D* ∈ℝ^*n*×*n*^ and *B* ∈ℝ^*m*×*m*^ are respectively the degree matrix and hyperedge matrix of hypergraph *G*, defined as:

(15) \begin{eqnarray*}{D}_{ii}& =\sum _{e=1}^{m}{W}_{ee}{H}_{ie}\end{eqnarray*}

(16) \begin{eqnarray*}{B}_{ee}& =\sum _{i=1}^{n}{H}_{ie}.\end{eqnarray*}

**Figure 3:**
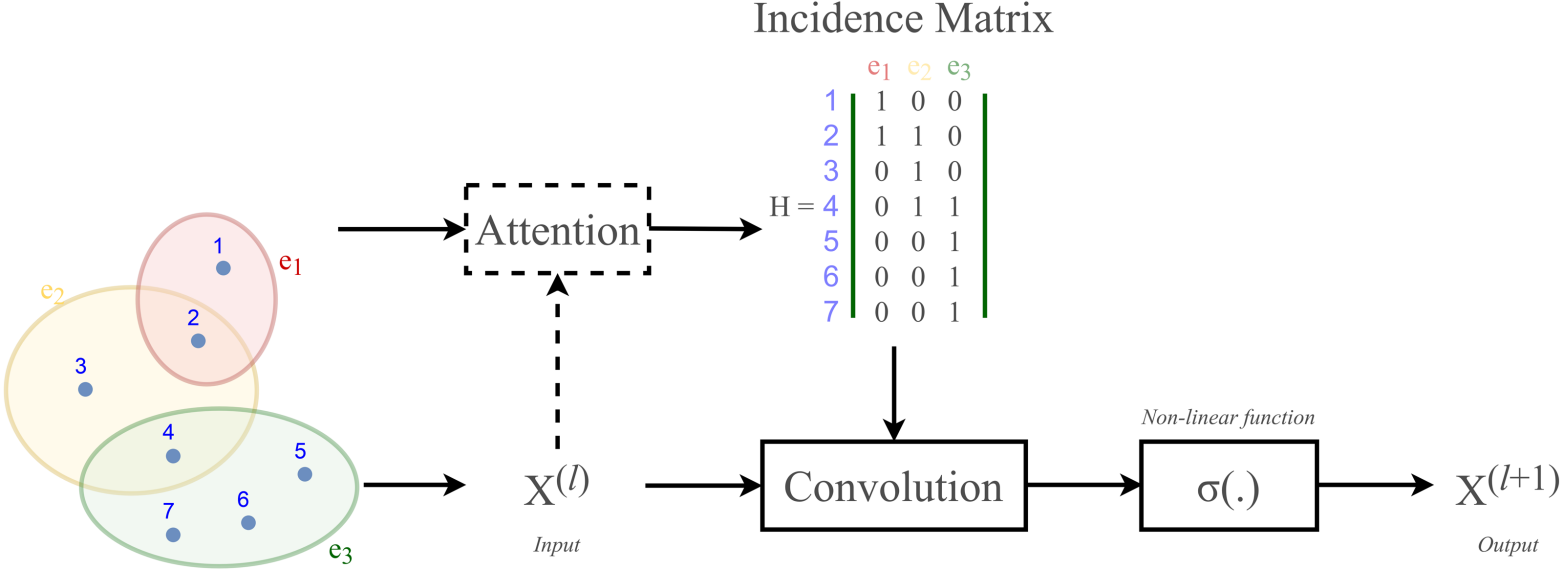
Schematic depiction of hypergraph convolution for a hypergraph of seven nodes and three hyper-edges.

According to Velickovic et al. (2018) and Lee et al. (2019), hypergraph convolution also owns a sort of attentional mechanism. However, such mechanism is not able to be learned nor trained in a graph structure (represented by the incidence matrix H). The purpose of attention in hypergraph is to learn a dynamic incidence matrix, followed by a dynamic transition matrix that can better reveal the intrinsic relationship between the nodes. Bai, Zhang & Torr (2021) suggested to solve this issue by applying an attention learning module on the matrix *H*. The overall demonstration of hypergraph convolution—hypergraph attention is displayed in Fig. 3 as follow.

### Related works

As presented in the first section, drug discovery is an essential purpose of the development of cheminformatics field. Specifically, significant tasks in cheminformatics that are now strongly supported by computational algorithms, especially deep learning, can include activity prediction, de novo generation, ADMET prediction, interaction prediction, and binding affinity prediction.

Activity prediction refers to a type of classification in which we study to see whether a specific drug has activity against one or more specific targets. A remarkable work in activity prediction using deep learning was presented by Wallach, Dzamba & Heifets (2015). In this research, Wallach used a deep CNNs model that could learn structure-based molecular data and succeeded in predicting active molecules against their chosen targets.

Differently, de novo generation basically aims at generating novel molecules. Leading in adopting GNNs for de novo generation, De Cao & Kipf (2018) introduced a graph learning model which was originally a GANs (Generative Adversarial Networks) framework operating on graph data. The model was proved to be able to generate diverse and novel molecules. For the same objective, Bresson & Laurent (2019) took advantage of graph auto encoder: they made GCNs layers to learn the representations of molecular data, next imposed them in a latent space, and then learned to reconstruct them back to graph data. Moreover, the graph generative model by Lim et al. (2019) successfully learned the scaffolds of molecules and constructed unseen molecules during learning.

Meanwhile, ADMET prediction is a drug discovery approach that can comprise of two path of resolution: predictive modeling, and generative modeling. In the scope of cheminformatics research, ADMET stands for *absorption, distribution, metabolism, elimination, and toxicity*, which are fundamental and crucial properties of drug candidates for their efficacy and safety in practical use. For ADMET prediction, firstly, we decide the molecular properties that we would like the drug to have. In predictive modeling, we aim at searching among existing compounds to find the ones that possess such properties. Suggested deep learning model by Feinberg et al. (2020) using graph convolutional neural networks had shown that GNN could learn from adjacency matrix and feature matrix of data to predict ADMET properties with higher accuracy compared to random forests and deep neural networks. Inversely, in generative modeling, computational models are made to generate molecules whose properties are matching the expected properties. A notable work in this problem was the research proposed by Segler et al. (2018).

Similar to activity prediction, ligand-protein interaction prediction is also a classification problem, but it takes the information of ligand and protein simultaneously. One of early researches in this approach is suggested by Gonczarek et al. (2018). Their deep learning architecture was utilized to learn structure-based data of molecules and proteins, and to predict molecule-protein interaction. Next, the end-to-end model which combined GNN and CNN introduced by Tsubaki, Tomii & Sese (2019) had proved to earn better evaluation results than various existing interaction prediction methods, such as a k-nearest neighbor, random forest, logistic regression, and support vector machine. In their model, GNN was applied to learn the ligands’ molecular fingerprints, and CNN was for proteins learning.

Lastly, binding affinity prediction is quite comparable to ligand-protein interaction prediction. This, however, is a regression problem that provides real values reflecting the *relationship* between ligands and proteins. It can be said that ligand-protein interaction prediction and binding affinity prediction are relatable because the affinity values can tell how *strong* the ligand-protein interaction is. In our article, binding affinity is alternatively referred as drug-target interaction (DTI). Current most studied learning methods for DTI prediction can be categorized into two approaches: supervised learning-based methods, for example: the study of Yamanishi et al. (2008), and semi-supervised learning-based methods, for which the research of Peng et al. (2017) is a remarkable work. One noticeable DTI prediction outcome came from the research of Karimi et al. (2019): in their DeepAffinity model, firstly, they engineered associate degree end-to-end deep learning model combining repeated neural networks (RNN) and convolutional neural networks (CNN) for learning representations of compounds and supermolecule targets, and for the prediction of compound-protein affinity; secondly, they expanded the model by using graph CNN, which is a basic form of GCN, to learn 2-D representations of compounds from SMILES strings. The latter model with GCN did beat the former one which was unified RNN-CNN when comparing results, and importantly it has shown that graph data and graph neural networks are potential for designing deep learning models and affinity prediction. Separately, other remarkable researches for DTI prediction using GNNs can include proposed work PADME (Feng et al., 2018) and GraphDTA (Nguyen et al., 2021). Experimental results from GraphDTA have shown superior performance of GNNs models over DeepDTA (Öztürk, Özgür & Ozkirimli, 2018) and WideDTA (Öztürk, Ozkirimli & Özgür, 2019) model, which are also well-known baseline *non-GNNs* cheminformatics researches that put the fundamental design for deep learning DTI prediction. The common characteristic of these four studies (PADME, DeepDTA, WideDTA, GraphDTA) is that they are all data-driven methods, which means their models are built to automatically learn the features of the inputs (Abbasi et al., 2020). Beside above frameworks where drugs and targets features are learned independently and simultaneously, research work of Li et al. (2021) suggested a new approach when they treated each pair of drug-target as one unit and learn the representation for each pair by GCN; the affinity values are predicted by a following deep neural network.

**Figure 4:**
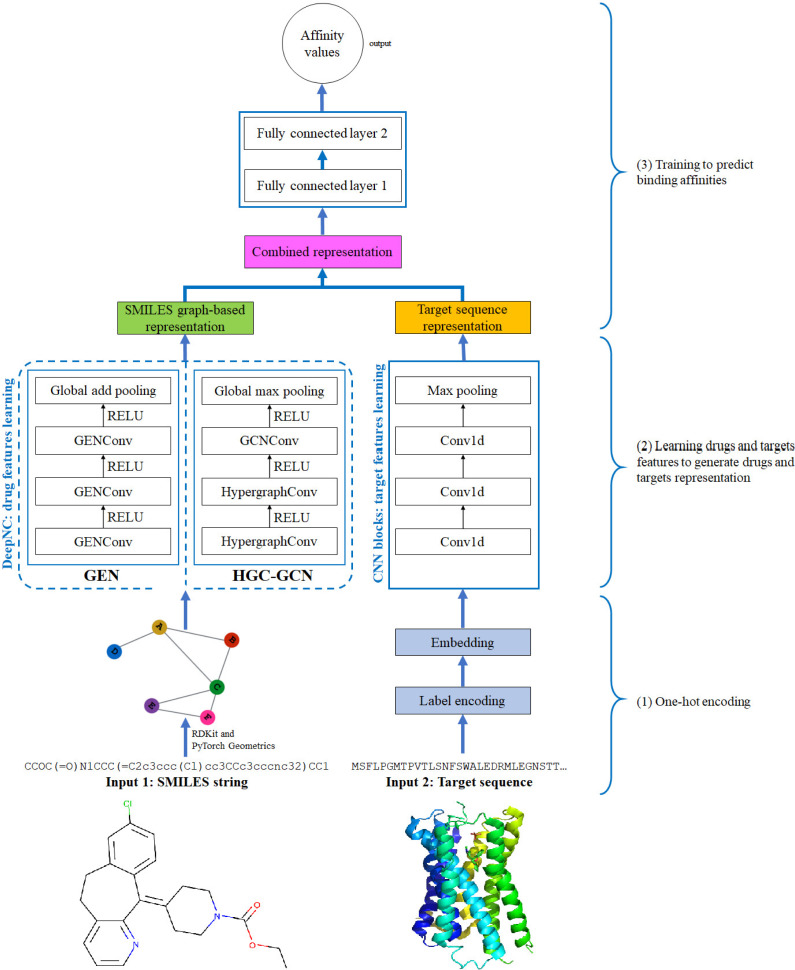
Diagram of proposed DeepNC architecture.

## Materials & Methods

### Design of DeepNC

In this section, we are going to explain our proposed model DeepNC whose primary components are visualized in detail in Fig. 4. Our model comprises of three main blocks: the first block (1)’s job is to translate the input data into the format that can be fed into the convolutional layers. For drugs, the input data is in form of SMILES strings and these strings will be converted into graphs containing information of the drug compounds features; meanwhile, the input targets which are initially in form of ASCII strings called target sequence will be embedded into vector representation for the next 1-D convolutional layers. Next, the second block (2) contains convolutional layers that will learn the drugs and targets features: GNNs layers learn drugs features to create drugs graph-based representation and at the same time, 1-D convolutional layers learn targets features to generate targets representation. In the third block (3), the representations are concatenated and fed into fully connected layers to calculate the binding affinity values. Details of each block will be discussed in the following sub-sections.

### Representation of drug

To connect chemistry language with computing language, we employ SMILES, which represents molecular compounds as ASCII characters strings, to be the format of input drugs data. SMILES stands for Simplified Molecular-input Line-entry System—a system that denotes chemical compounds as line notation: molecules are described in alphabets and characters.

The SMILES strings of drug compounds will be converted into molecular graphs which contains important features of drugs and which is the data format that can be fed into GNNs layers. Each molecular graph has to have these information: the number of atoms (of the compound), atom features and edge index. In graph-learning language, edges represent the bonds between atoms and the atoms are alternatively called nodes. Atom features of a molecule is a set of features describing a node in the graph. In this study, we used five classes of information to demonstrate atom features: the atom symbol (symbols that are present in the SMILES strings), the number of adjacent atoms, the number of adjacent hydrogen, the implicit valence of the atom and whether the atom is in aromatic structure. In order to create molecular graphs from SMILES for learning tasks, we employed RDKit (RDKit, 2022) and PyTorch (Paszke et al., 2017).

### Representation of target

In studied drug-target datasets, each target is demonstrated in a protein sequence. Such sequence is an ASCII string representing amino acids and is obtained from UniProt database (Jain et al., 2009) using the target’s gene name. Specifically, there are 20 amino acids in nature which contribute to create a protein.

### Drug-target interaction

Interaction between a drug and a target can be recognized by the value of binding affinity. Binding affinity is defined as a measurement that can be used to estimate the strength of the interaction between a single biomolecule and its binding partner, which is also termed as ligand. It can be quantified, providing information on whether or not molecules are interacting as well as assigning a value to the affinity. Typically, when measuring binding affinity, researchers are interested in several parameters, but mostly in the unit of measurement called the dissociation constant (K_d_), which defines the likelihood that an interaction between two molecules will break (Gilson, 2010).

### Graph convolutional layers

The proposed DeepNC framework includes two variants namely GEN and HGC-GCN, as shown in the diagram in Fig. 3. The variant GEN contains three GENConv layers and one global add pooling layer, while the variant HGC-GCN contains 2 HypergraphConv layers, one GCNConv layer and one global max pooling. These layers are used to produce graph-based representation of input drugs.

**Figure 5:**
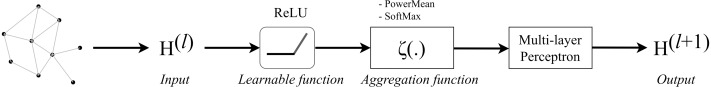
Diagram of a GENConv layer.

GENConv is a generalized graph convolution layer adopted from the research work (Li et al., 2020) which has earlier been mentioned in Generalized Aggregation Graph Network. From formula Eqs. (7) and (11), we simplified the message construction function as:

(17) \begin{eqnarray*}{x}_{i}^{{^{\prime}}}=MLP({x}_{i}+AGG\{ (RELU({x}_{j}+{e}_{ij})+{|}j\in N(i)\} ))\end{eqnarray*}

where *MLP* (⋅) is a multi-layer perceptron and the aggregation scheme to use is *softmax_sg*. The global add pooling layer in GEN returns batch-wise graph-level-outputs by adding node features across the node dimension. The GENConv layer can be depicted by Fig. 5.

**Table 1:** Summary of studied datasets.

**Datasets**	**Number of targets**	**Number of compounds**	**Number of interaction pairs**
Davis	442	68	30056
Kiba	229	2111	118254
Allergy	35	286	372

HypergraphConv is a hypergraph convolutional operator adopted from the research work (Bai, Zhang & Torr, 2021) which has been explained in Hypergraph Convolution and Hypergraph Attention. The operator is simplified from Eq. (13) and presented as:

(18) \begin{eqnarray*}{X}^{{^{\prime}}}={D}^{-1}HW{B}^{-1}{H}^{T}X\Theta \end{eqnarray*}

where *H* ∈{0, 1}^*N*×*M*^ is the incidence matrix, W ∈ℝ^*M*^ is the diagonal hyperedge weight matrix, and and *D*, *B* are the corresponding degree and hyperedge matrices. An attention will be added to this layer. Illustration of a HypergraphConv layer has been shown in Fig. 3.

GCNConv is a graph convolutional layer extracted from the research (Kipf & Welling, 2017). The operator of the layer is written as:

(19) \begin{eqnarray*}{X}^{{^{\prime}}}={\hat {D}}^{- \frac{1}{2} }\hat {A}{\hat {D}}^{- \frac{1}{2} }X\Theta \end{eqnarray*}

### Datasets

Our proposed model is evaluated on two different datasets: Davis and Kiba. Numbers of compounds and targets of each dataset are noted in Table 1. Davis is a Kinase dataset that was introduced by Davis et al. (2011), containing 25,046 pairs of drug-target having binding affinities measured as K_d_ with values ranging from 5.0 to 10.8 (nM). Meanwhile, Kiba dataset was contributed by Tang et al. (2014), and it has binding affinities measured as Kiba score with values ranging from 0.0 to 17.2. The SMILES strings of compounds from both datasets were originally obtained from the PubChem compound database (Wang et al., 2017) based on their PubChem CIDs and their protein sequences were extracted from the UniProt protein database.

Beside two above baseline datasets, we are building a potential dataset called ‘allergy drugs’ dataset and also involving it in training and evaluating DeepNC. This dataset are formed by firstly investigating and collecting the drugs which are used to treat allergic reactions, which are referred as ‘allergy drugs’ in this research, and their respective targets; the list of allergy drugs and their targets is achieved from DrugBank; secondly, finding and aggregating the ligands that have interactions with the allergy drug targets and noting down together with their K_d_ values. Specifically, chosen ligand-target pairs have K_d_ ranging from 1.0 to 15.0 (nM). SMILES strings of compounds and K_d_ values were extracted from BindingDB database and the target sequences were extracted from the UniProt protein database based on accession numbers. Motivated by the aim of searching for medication solutions for allergies treatment, this proposed dataset is our effort in contributing in the research and discovery of allergy drugs. Summarized information of this independent dataset is also noted in Table 1.

### Evaluation metrics

Two metrics that are used to evaluate the model performance are mean square error (MSE) and concordance index (CI). MSE reflects the difference between the predicted values and the expected (actual) values. During training, a learning model attempts to reduce the gap between the actual value and the prediction. We used MSE as the loss function because we are working on a regression problem, where *P* is the prediction vector and *Y* is the vector of actual *n* outputs. The number *n* denotes the sample size. MSE is determined using the following formula:

(20) \begin{eqnarray*}MSE= \frac{1}{n} \sum _{i=1}^{n}({P}_{i}-{Y}_{i})^{2}\end{eqnarray*}

In order to state whether the order of a predicted value of two random drug-target pairs is identical to the order of the true value, we use the Concordance Index (CI). The calculation of CI is in accordance with (21)

(21) \begin{eqnarray*}CI= \frac{1}{Z} \sum _{{\delta }_{i}\gt {\delta }_{j}}h({b}_{i}-{b}_{j})\end{eqnarray*}

where *b*_*i*_ is the predicted value for the larger affinity *δ*_*i*_, *b*_*j*_ is the predicted value for the smaller affinity *δ*_*i*_, *Z* is a normalization constant, *h* (*x*) is the step function presented as:

(22) \begin{eqnarray*}h(x)= \left\{ \begin{array}{@{}l@{}} \displaystyle 1 \mathrm{if} x\gt 0\\ \displaystyle 0.5 \mathrm{if} x=0\\ \displaystyle 0 \mathrm{if} x\lt 0 \end{array} \right. \end{eqnarray*}

**Table 2:** Parameters setting for DeepNC models.

***Parameters***	***Settings***
Learning rate	0.0005
Batch size	256
Epoch	1000
Optimizer	Adam
Graph convolutional layers in GEN	3
Graph convolutional layers in HGC-GCN	3

**Figure 6:**
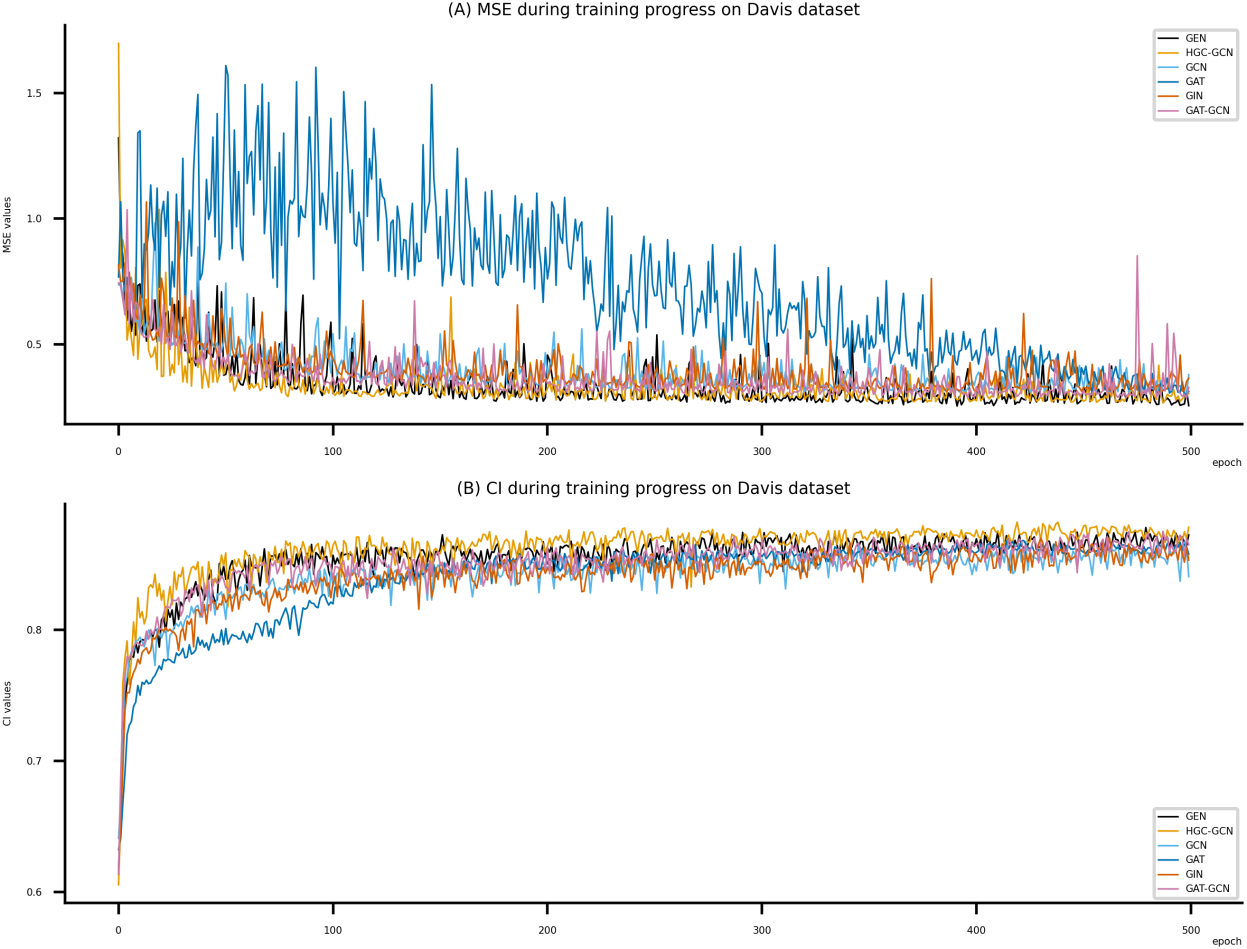
Performance of DeepNC and GraphDTA training models on the Davis dataset. (A) MSE Values, (B) CI Values.

**Figure 7:**
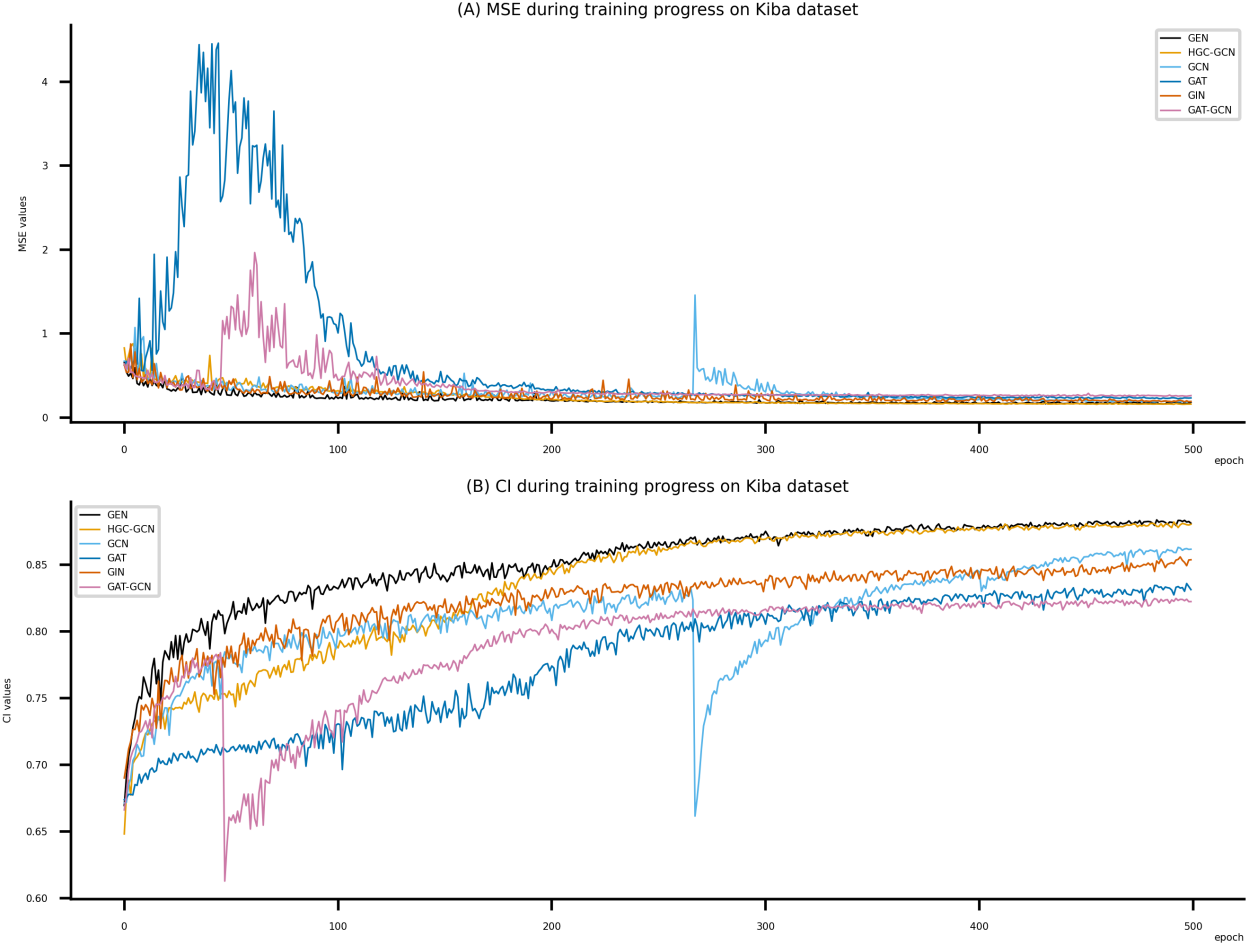
Performance of DeepNC and GraphDTA training models on the Kiba dataset. (A) MSE Values (B) CI Values.

**Figure 8:**
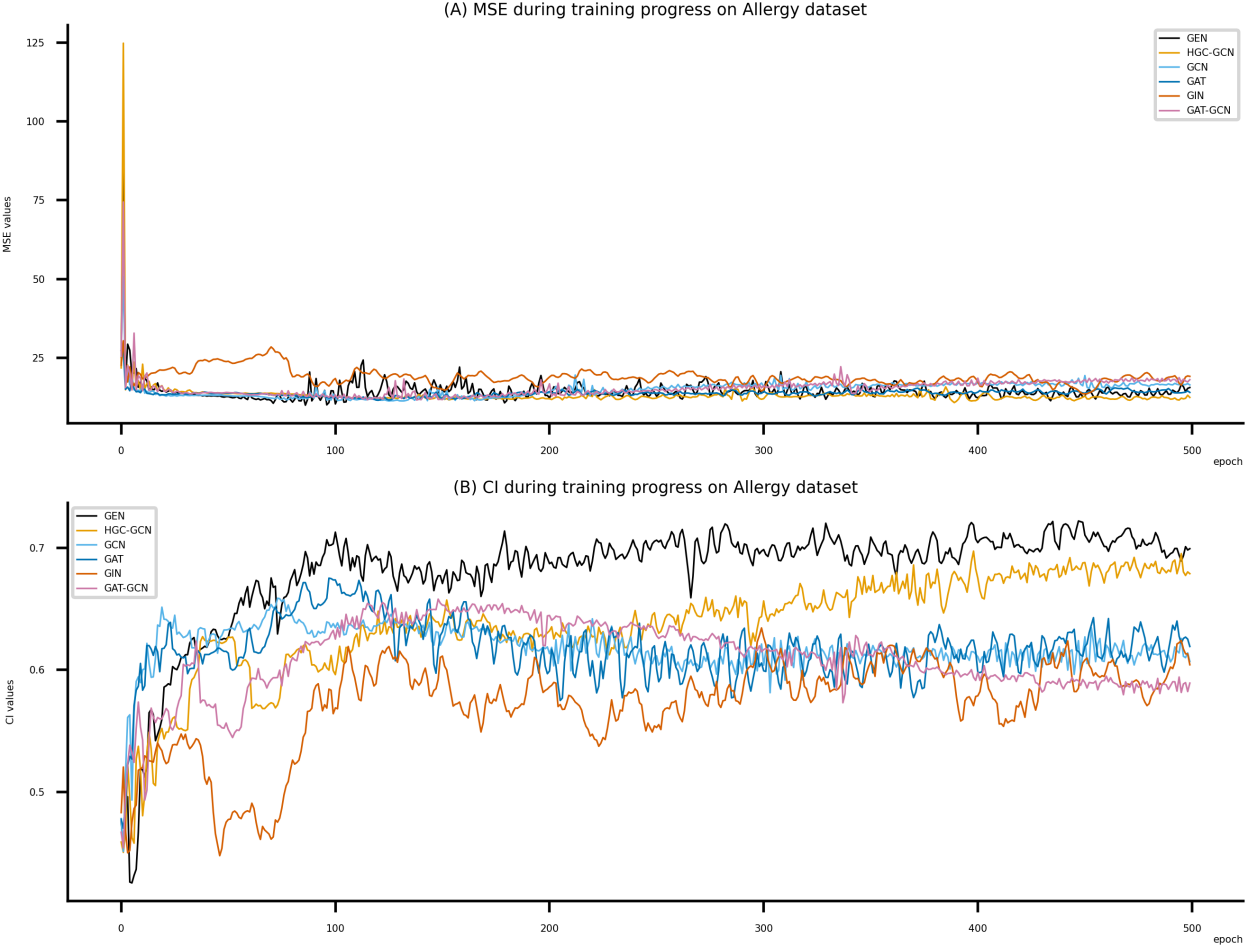
Performance of DeepNC and GraphDTA training models on the Allergy dataset. (A) MSE Values (B) CI Values.

**Figure 9:**
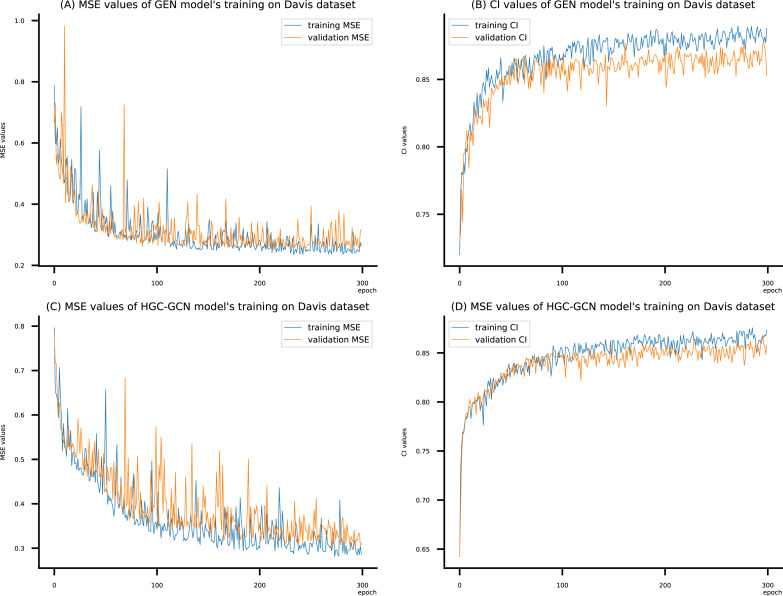
(A–D) DeepNC models’ performance on the Davis dataset.

**Figure 10:**
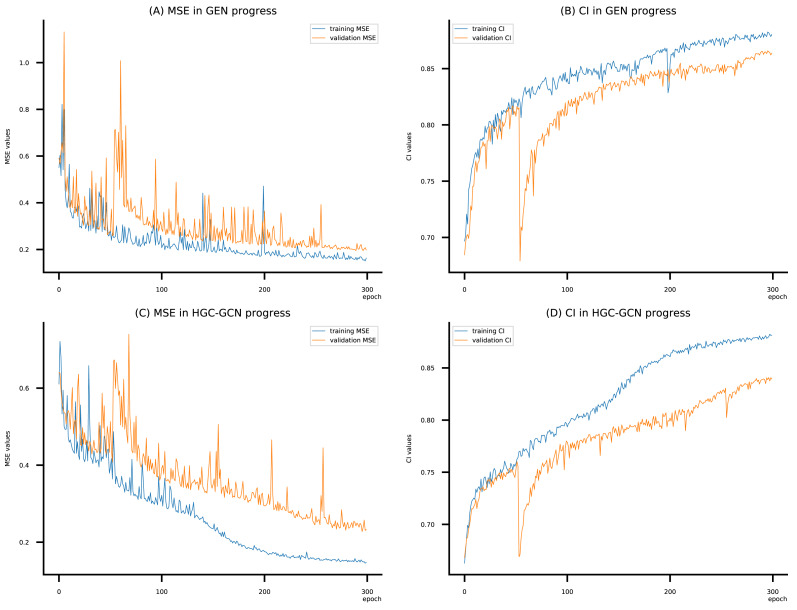
(A–D) DeepNC models’ performance on the Kiba dataset.

**Figure 11:**
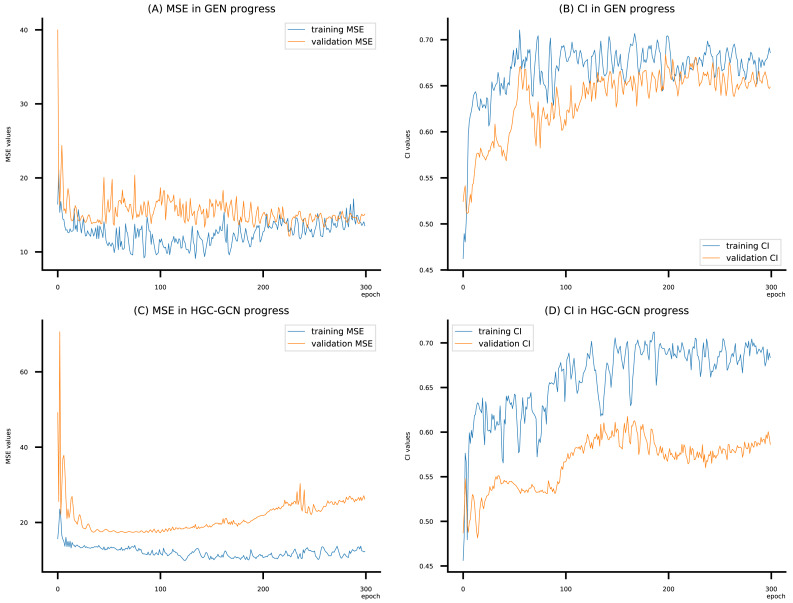
(A–D) DeepNC models’ performance on the Allergy dataset.

### Training settings

Hyperparameters applied on the GNN variants used in the training of DeepNC are summarized in Table 2. The learning has performed with 1,000 epochs, and the network’s weights were updated using a mini-batch of size of 512. To train the networks, Adam have employed as the optimization algorithm. With the default learning rate of 0.0005. Table 2 summarizes the settings for the model trainings.

## Results

### Models’ training progress charts

We utilized the MSE and CI values to assess the performance of proposed models in DeepNC and to compare it with the current state-of-the-art methods Simboost (He et al., 2017), DeepDTA (Nguyen et al., 2021) and GraphDTA (Öztürk, Özgür & Ozkirimli, 2018), which we chose as baselines. As mentioned in Related works, GraphDTA has improved the performance of models from DeepDTA and WideDTA by replacing the CNN layers for drug representation with GNN layers. Accordingly, our research is not only meant to outperform non-GNN methods (SimBoost, DeepDTA) but is aiming at enhancing current GNN models (GraphDTA) as well.

Figsures 6–8 display the values of MSE and CI of each epoch being trained by DeepNC (GEN, HGC-GCN) and GraphDTA (GCN, GAT, GIN, GAT-GCN), observed on three datasets.

On each figure with 2 charts, we notice that MSE and CI values of GEN and HGC-GCN in general are better than those values of the other four models, *i.e.*, the MSE is smaller (when MSE line is lower) and CI is larger (when CI line is higher).

To compare the training of DeepNC models with and without validation, the results are presented in Figs. 9–11. It should be noted that trainings without validation are conducted on the *train* set of each dataset and then the models are used to predict on the *test* set. Meanwhile, trainings with validation starts with models being trained on 80% of the *train* set and after that the models are used to prediction on the rest 20%, which is referred as the *valid* set. For training with validation by each model, the *valid* set is randomly split from the *train* set when we run the Python program.

### Evaluation results

Experimental results of DeepNC and the baseline methods are remarked in Tables 3–7. We compare our models to SimBoost (He et al., 2017) (non-deep learning method), DeepDTA (Öztürk, Özgür & Ozkirimli, 2018) (non-GNN deep learning) and GraphDTA (Nguyen et al., 2021) (deep learning with GNN architecture). It should be noted that results are extracted from the models’ training on the test sets. For Davis dataset, training results are noted in Tables 3 and 4. Tables 5 and 6 contain results for training with Kiba dataset.

Tables 3 and 5 show MSE and CI values of models being trained on Davis and Kiba dataset. Meanwhile, Tables 4 and 6 report the values of ${r}_{m}^{2}$ which evaluate the external predictive performance of QSAR models, in which ${r}_{m}^{2}$ >0.5 for the test set means that the models are determined to be acceptable.

For the independent dataset Allergy, we only reported training results by MSE and CI values in Table 7.

For all datasets, noted results show that GEN and HGC-GCN have smaller MSE values, and larger CI values than those of the benchmark models. In terms of MSE, the results suggest that model GEN of DeepNC performed better than SimBoost, DeepDTA (*p*-value of 0.008 for both) and GraphDTA (*p*-value of 0.002); and model HGC-GCN of DeepNC performed better than SimBoost (*p*-value of 0.004), DeepDTA (*p*-value of 0.016) and GraphDTA (*p*-value of 0.002) on Davis dataset. Similarly, on Kiba dataset, the performance results suggest that model GEN of DeepNC predicted better than SimBoost (*p*-value of 0.002), DeepDTA (*p*-value of 0.004) and GraphDTA (*p*-value of 0.0001); and model HGC-GCN performed better than SimBoost (*p*-value of 0.062), DeepDTA ( *p*-value of 0.098) and GraphDTA (*p*-value of 0.016).

## Discussion

In this work, we propose a framework method for predicting drug-target binding affinity, called DeepNC which represents drugs as graphs. Using deep convolution networks on GNN algorithms show that DeepNC can predict the affinity of drugs-targets better than not only non-GNN deep learning methods such as SimBoost and DeepDTA, but also GNN method (GraphDTA) and shown significant improvements over thoses methods. DeepNC perform consistently well across two separate benchmark databases in MSE, CI performance measures. Table 3 grants the performance on method, target representation, drug representation in MSE and CI for various approaches to predict the Davis dataset. The best MSE value from the baseline methods is 0.261 by DeepDTA, for both drugs and proteins are represented as 1D strings. Training on the same dataset by DeepNC, for MSE values, the model GEN has gained 0.233 and HGC-GCN gained 0.243, which has improved ±0.028 of MSE and improved ±0.009 of CI when compared to DeepDTA.

In Table 5, we observed results of the larger Kiba dataset. The baseline method of GraphDTA’s GCN has the MSE of 0.185 and CI of 0.862. The proposed GEN from DeepNC has outperformed by values of MSE and CI which are 0.133 and 0.897 respectively. Those results given by HGC-GCN are 0.172 and 0.872. Hence, we noticed that DeepNC has provided better result of MSE by ±0.048 and CI by ±0.035.

Beside the testing on benchmark datasets, we have experimented with the Allergy dataset. Here we consider Graph attention networks (GAT), Graph Isomorphism (GIN), GAT-GCN, and GCN from baseline GraphDTA with GCN shows the best MSE (9.312) and the best CI (0.693). From Table 7, our proposed DeepNC, as compared with GraphDTA, has shown improvement of GEN by MSE of 9.095 and CI of 0.699, and improvement of HGC-GCN by MSE of 9.915 and CI of 0.722. Briefly, DeepNC has improved ±0.217 for MSE and ±0.029 for CI.

From the results, it is suggested that representing molecules as graphs improves the performance considerably and with the combination of GEN and HGC-GCN of the framework confirm deep learning models for graphs are appropriate for drug-target interaction prediction problem.

**Table 3:** MSE and CI values of models’ training on the Davis dataset.

Method	Model	Drugs rep. (learning method)^a^	Targets rep. (learning method)^a^	MSE	CI
SimBoost		PubChem Sim	S-W	0.282	0.872
DeepDTA		SMILES (CNN)	S-W	0.420	0.886
		SMILES (CNN)	Target sequence (CNN)	0.261	0.878
	GCN	Graph (GCN)	Target sequence (CNN)	0.302	0.859
GraphDTA	GAT	Graph (GAT)	Target sequence (CNN)	0.295	0.865
	GIN	Graph (GIN)	Target sequence (CNN)	0.308	0.860
	GAT_GCN	Graph (combined GAT-GCN)	Target sequence (CNN)	0.286	0.870
DeepNC	GEN	Graph (GEN)	Target sequence (CNN)	0.233	0.887
	HGC_GCN	Graph (HGC-GCN)	Target sequence (CNN)	0.243	0.881

**Notes.**

a The method of learning drug/target features are given in parenthesis.

## Conclusion

Graph-based learning neural network models are worth studying in terms of generating molecular graphs, because the direct use of graphs has many advantages that character string representation does not have is first, and most importantly, each molecular subgraph is interpretable. DeepNC, a new GNN molecular design framework, was described and utilized to explore novel graph-based topologies for molecular generation in this study. Deep Neural Computing (DeepNC), a new deep learning-based framework for DTI prediction that uses three GNN algorithms, is our suggested framework. Here, DeepNC demonstrated the molecular graph context tailored for drug target interaction models, where three different GNNs have investigated: Generalized Aggregation Networks (GEN), Graph Convolutional Networks (GCN), and Hypergraph Convolution-Hypergraph Attention (HGC). Hypergraphs provide a flexible and natural modeling tool to model such complex molecule structure. HypergraphConv estimates each hyperedge of the hypergraph by a customary of pair of edges connecting the vertices of the hyperedge and gives the learning problem as a graph-learning problem. The DeepNC outperforms all other models in terms of both speed and quality of generated prediction structures. Attention with graph neural network able to expand an additional flexible model and will applied to a variety of applications at the same time as hypergraph convolution and hypergraph.

**Table 4:** The average r^2^ scores of models’ training on the Davis dataset.

Method	Model	Drugs rep. (learning method)^a^	Targets rep. (learning method)^a^	${r}_{m}^{2}$
SimBoost		PubChem Sim	S-W	0.644
DeepDTA		SMILES (CNN)	Target sequence (CNN)	0.630
DeepNC	GEN	Graph (GEN)	Target sequence (CNN)	0.653
	HGC_GCN	Graph (HGC-GCN)	Target sequence (CNN)	0.686

**Notes.**

a The method of learning drug/target features are given in parenthesis.

**Table 5:** MSE and CI values of models’ training on the Kiba dataset.

Method	Model	Drugs rep. (learning method)^a^	Targets rep. (learning method)^a^	MSE	CI
SimBoost		PubChem Sim	S-W	0.222	0.836
DeepDTA		SMILES (CNN)	S-W	0.204	0.854
		SMILES (CNN)	Target sequence (CNN)	0.194	0.863
	GCN	Graph (GCN)	Target sequence (CNN)	0.185	0.862
GraphDTA	GAT	Graph (GAT)	Target sequence (CNN)	0.223	0.834
	GIN	Graph (GIN)	Target sequence (CNN)	0.186	0.852
	GAT_GCN	Graph (combined GAT-GCN)	Target sequence (CNN)	0.253	0.824
DeepNC	GEN	Graph (GEN)	Target sequence (CNN)	0.133	0.897
	HGC_GCN	Graph (HGC-GCN)	Target sequence (CNN)	0.172	0.872

**Notes.**

a The method of learning drug/target features are given in parenthesis.

**Table 6:** The average scores of models’ training on the Kiba dataset.

Method	Model	Drugs rep. (learning method)^a^	Targets rep. (learning method)^a^	${r}_{m}^{2}$
SimBoost		PubChem Sim	S-W	0.629
DeepDTA		SMILES (CNN)	Target sequence (CNN)	0.673
DeepNC	GEN	Graph (GEN)	Target sequence (CNN)	0.695
	HGC_GCN	Graph (HGC-GCN)	Target sequence (CNN)	0.624

**Notes.**

a The method of learning drug/target features are given in parenthesis.

**Table 7:** MSE and CI values of models’ training on the Allergy dataset.

Method	Model	Drugs rep. (learning method)^a^	Targets rep. (learning method)^a^	MSE	CI
GraphDTA	GCN	Graph (GCN)	Target sequence (CNN)	9.312	0.693
	GAT	Graph (GAT)	Target sequence (CNN)	11.200	0.661
	GIN	Graph (GIN)	Target sequence (CNN)	12.158	0.659
	GAT_GCN	Graph (combined GAT-GCN)	Target sequence (CNN)	9.951	0.683
DeepNC	GEN	Graph (GEN)	Target sequence (CNN)	9.095	0.699
	HGC_GCN	Graph (HGC-GCN)	Target sequence (CNN)	9.159	0.722

**Notes.**

a The method of learning drug/target features are given in parenthesis.

## Supplemental Information

10.7717/peerj.13163/supp-1 Supplemental Information 1 Supplemental Material

10.7717/peerj.13163/supp-2 Supplemental Information 2 The distribution of binding affinity values of three datasets

## Additional Information and Declarations

### Data Availability

The following information was supplied regarding data availability: The data and models are available at GitHub: https://github.com/thntran/DeepNC.
