# Supplementary material for: DeepNC: a framework for drug-target interaction prediction with graph neural networks
Source: PeerJ. 2022 May 11;10:e13163. doi: 10.7717/peerj.13163 (PMC9107302; doi:10.7717/peerj.13163)
Supplement: Supplemental Information 1 [file peerj-10-13163-s001.docx]

**Supplement to:** **DeepNC: A Framework for Drug-Target Interaction Prediction with Graph Neural Networks**

**1. Pseudocode for DeepNC**

Each node (v_i_) in $\mathcal{G}_{\mathcal{N}}$has been assigned a rank Algorithm 1. The breadth-first search algorithm can help with ranking method. Let $V_{\mathcal{N}}$ be the graph visited node and the order can be stepwise until there is no more graph nodes, or repeat until all graphs (molecule SMILES) have been input as batches. We use RDkit [1] and PyTorch [2] parameters for the input construction

**Algorithm 1:** Pseudocode for reading graph layer

**Input:** Graph$\mathcal{G}\left( \mathcal{V,ℇ} \right);$Molecule to Graph (molecule);

a molecule s in SMILES catalogue C

atom vector with fixed length m, vector distance c.

**Output:** for all $v \epsilon\mathcal{V;}$

1. Visit_ nodes = a list of nodes have been visited (v_i_)

2. **While** |visited_nodes| < |$\mathcal{V}$ | **do**

new neighbour_nodes = $\mathcal{N}$(visited_node) – visited_node;

3. **While** |new_neighbour_nodes| > 0 **do**

V_j_ = **argmin** (new_neighbour_nodes);

4. nodes_visited.append(V_j_);

new_neighbour_nodes.delete/remove(v_j_)

5. $\mathcal{V}$ = |visted_nodes.index(v_i_)

**return** ($\mathcal{V) G}$

We investigate the performance of GCN on large molecular graphs in this study. We present the innovative Generalized Aggregation Function for graph convolutions to improve the effect of aggregation functions. The function is fully differentiable and can be taught in a process manner using edge analysis. We also consider the impact of hidden neurons in a variety of graph datasets. We can improve the performance in the Davis, Kiba, and independent allergy datasets by implementing a novel graph normalising layer. The subsequent paragraph discusses on the neural message passing.

The use of neural message transmission, in which vector messages are transferred between nodes on the graph and modified by the neural network, is the distinguishing feature of GNN. Algorithm 2 handles the message transmission of single node message aggregation across the neighbour node molecular graph structure from the line 1 to line 6. Input of generalized aggregation message set$\left\{ m_{vu}|u \epsilon\mathcal{N}\left( v \right)\} \right.$the nodes $m_{vu}\epsilon\mathbb{R}^{D}$*SoftMax_Agg* $(\cdot)$ for all edges for mean aggregation. The $aggregate$ function takes the set of embedding is of the nodes graph neighbourhood $\mathcal{N}\left( v \right)$as input and generates a message based on aggregated neighbourhood information at each iteration of the GNN where update and aggregate are arbitrary neural network functions.

**Algorithm 2:** Pseudocode for GEN and HGC-GCN

**Input:** Graph$\mathcal{G}\left( \mathcal{V,ℇ} \right);$node structures (molecule) $\left\{ x_{v} \right.,\forall_{\mathcal{V}}\epsilon\mathcal{V\};}$

Embed of layers $\mathcal{K}$: Message passing layer $\left\{ m_{vu}|u \epsilon\mathcal{N}\left( v \right)\} \right.$

Identify the nodes $m_{vu}\epsilon\mathbb{R}^{D}$*SoftMax_Agg* $(\cdot)$ for all nodes

edge detection features.

**Output:** Node embedding for all $v \epsilon\mathcal{V;}$

**1**: **for** $v \epsilon\mathcal{V}$ **do**

**2**: $h_{u}^{\left( k+1 \right)}$ = update ^(k)^ $\left( h_{u}^{\left( k \right)}, {aggregate}^{\left( k \right)}\left( \left\{ h_{v}^{\left( k \right)} \right., \forall_{v} \in\mathcal{N}\left( u \right) \right\} \right)$

**3**: **for** *k =*1,…….*K* **do**

**4:** **for** $u\in\mathcal{G}_{v}^{(K)}$ **do**

**5**: $h_{u}^{(k)}\leftarrow{aggregate}^{(k)}$ = ReLU($h_{v}^{(l)}+ m_{v}^{(l)})$

**6:**  $h_{v}\leftarrow h_{v}^{(K)}$

**7: for** Each ***hyperedge*** $\epsilon\in E= W_{\in\in}$ weights of diagonal matrix

$W \epsilon\mathbb{R}^{MxM}$ **do**

**8: for** $\mathcal{H}=(V,E)$ **do** |V| = n, |E| =m *hypernodes* set V_L_

***hypernode*** $\mathcal{V}\in V= \left\{ 1, \right.\ldots, n\}$

**9:**  **for** $h_{v}:v \in V$ **do**

Feature vector $x_{v}\epsilon\mathbb{R}^{p}$, $X \in\mathbb{R}^{n \times p}$

*predict* labels set V\V_L_

**10:** Compute prediction $\hat{Y_{t}} \leftarrow f(\mathcal{G}^{t-1})$

**11:** Assign labels to unlabeled nodes in graph

**12:** Amend graph $\mathcal{G}^{0}$ by add or remove inter-class and edges.

**13:** return: new predict graph $\mathcal{G}\left( \mathcal{V,ℇ} \right);$

A good graph model for describing a complex relationship is the hypergraph defined in Line 7, where a hyperedge can connect more than two vertices. For example, consider the hypergraph (V,) which has N vertices and M hyper-edges. Each hyperedge has given a positive weight in the loop in Line 7, and all of the weights were recorded in a diagonal matrix. In terms of research methods, Hypergraph convolution approximates each hyperedge of the hypergraph with a set of pairwise edges connecting the hyperedge's vertices, and the learning issue have treated as a graph-learning problem on the approximation. Line 10 extend the computation of prediction, and return the new molecule predicted graph affinity structures.

**2. Example of SMILES for drug representation**

For example, c and C represent aromatic and aliphatic carbon atoms, O denotes oxygen, and the symbols −, =, and # represent single, double, and triple bonds between atoms of a molecule. To indicate rings, a number is located at the two atoms where the ring is closed. For example, benzene (C_6_H_6_) in aromatic SMILES notation would be written as c1ccccc1. Usually, round brackets are used to denote side chains.

**3. Example of protein sequence for target representation**

In the label encoding phase, each protein sequence will be encoded with an integer based on the alphabetical order of its associated symbol, e.g., Alanine (A) is 1, Cystine (C) is 3, Aspartic Acid (D) is 4, Glutamic acid (E) is 5, and so on, until Tyrosine (Y) is 24, allowing the initial ACSII sequence to be presented as an integer sequence. The length of each integer sequence is fixed at 1000. Shorter sequences will be padded with zero values and longer sequences will be truncated. The label encoding for the example sequence of amino acids ‘VFRRDA’ is given below:

[V F R R D A] = [21 6 17 17 4 1]

Integer sequences will then be taken as input to embedding layers which return a 128-dimentional vector representation for the next 1-D convolutional layers.

**4. Distribution of binding affinity values of datasets**

Distribution of binding affinity values of the drug-target pairs for each dataset is illustrated in Fig. 1.

**Figure 1. The distribution of binding affinity values of three datasets.**

**5. Activation function**

The choice of activation function can significantly impact performance of networks. One important activation function which has been widely used in graph neural networks is the rectified linear unit activation function.

$\mathrm{ReLU}(\mathrm{x})=\left\{\begin{matrix} x\text{ if }x>0 \\ 0\text{ otherwise} \end{matrix}\right.$ (1)

The idea behind ReLU is to encourage a network to “turn off” some hidden units, yielding a sparser signal.

**6. Software and hardware**

All model implementations are conducted in Python programming language. The most important Python library utilized in the whole DeepNC project is the framework PyTorch [2] and its extension PyTorch Geometric. PyTorch is an optimized tensor library for deep learning using GPUs and CPUs; it essentially consists of a collection of neural network building blocks that keep track of their own gradients, a collection of gradient descent-based optimizers and support for GPU acceleration.

In addition, the PyTorch Geometric extension aids in the creation of GNN models. Geometric deep learning is a collection of approaches for deep learning on graphs and other irregular structures. A simple mini-batch loader for numerous tiny and one gigantic graphs, a large number of common benchmark datasets (based on simple APIs to construct your own), and useful transforms, both for learning on arbitrary graphs and 3D meshes or point clouds The cheminformatics library RDKit, on the other hand, is used to manage molecular data. Specifically, it turns SMILES strings of compounds into graphs and generates atom feature vectors. The training of models is carried out on a GPU, which is an NVIDIA GeForce RTX 2080 Ti.

**Reference**

1. RDKit: Open-source cheminformatics. URL: <https://www.rdkit.org>
2. Adam Paszke et al. “Automatic differentiation in PyTorch”. In: *NIPS-W*. 2017
