# Supplementary material for: DeepNC: a framework for drug-target interaction prediction with graph neural networks
Source: PeerJ. 2022 May 11;10:e13163. doi: 10.7717/peerj.13163 (PMC9107302; doi:10.7717/peerj.13163)
Supplement: Supplemental Information 2 [file peerj-10-13163-s002.pdf]

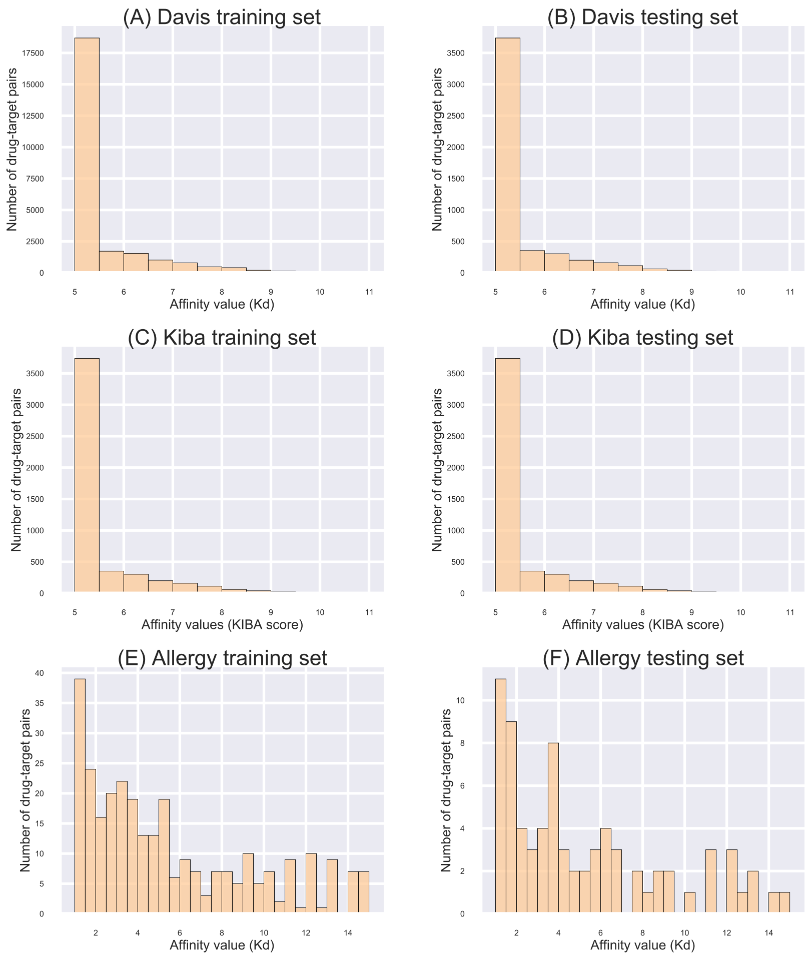

(A) Davis training set
Number of drug-target pairs
0
2500
5000
7500
10000
12500
15000
17500
5
6
7
8
9
10
11
Affinity value (Kd)
(B) Davis testing set
Number of drug-target pairs
0
500
1000
1500
2000
2500
3000
3500
5
6
7
8
9
10
11
Affinity value (Kd)
(C) Kiba training set
Number of drug-target pairs
0
500
1000
1500
2000
2500
3000
3500
5
6
7
8
9
10
11
Affinity values (KIBA score)
(D) Kiba testing set
Number of drug-target pairs
0
500
1000
1500
2000
2500
3000
3500
5
6
7
8
9
10
11
Affinity values (KIBA score)
(E) Allergy training set
Number of drug-target pairs
0
5
10
15
20
25
30
35
40
2
4
6
8
10
12
14
Affinity value (Kd)
(F) Allergy testing set
Number of drug-target pairs
0
2
4
6
8
10
2
4
6
8
10
12
14
Affinity value (Kd)
